# Supplementary material for: The Chromatin Protein CFDP1 Activates TPX2 and Promotes Chromosomal Microtubule Nucleation and Spindle Assembly
Source: Int J Mol Sci. 2026 Jan 29;27(3):1362. doi: 10.3390/ijms27031362 (PMC12898535; doi:10.3390/ijms27031362)
Supplement: Supplementary file 1 [file ijms-27-01362-s001.zip › Supplementary Figures Legends.pdf]

Figure legends for Supplementary Figures

Figure S1. Targeting constructs and knockout strategy for *Cfdp1* knockout and conditional knockout mice. (**A**) Schematic detailing targeting vector construction for the generation of *Cfdp1* knockout mice. Exon 1 is replaced by a *LacZ-Neo* cassette in targeted mice. (**B**) Southern blot analysis of targeted embryonic stem cells (upper) and genotyping PCR confirmation of *Cfdp1* targeted mice (lower). (**C**) Embryonic assessment of lethality in *Cfdp1* knockout embryos. (**D**) Targeting vector design for the generation of *Cfdp1* conditional knock out mice. Exon 1 is flanked by *flox* sites and can be excised by CRE expression. (**E**) Southern Blot verification of the targeting construct and genotyping PCR confirmation of conditional knockout mice.

Figure S2. CFDP1 cell fractionation, localization and chromosome segregation defects in CFDP1 depleted cells. (**A**) Immunoblot for CFDP1 levels in cytosolic, nuclear and chromatin enriched fractions prepared from NIH3T3 cells. (**B**) Immunoblot analysis for CFDP1 in G2/M phase arrested NIH3T3 mitotic chromosome fraction, corresponding supernatant and the whole cell lysate. (**C**) Immunofluorescence analysis demonstrating CFDP1 localization within the nucleus. CFDP1 (red) is targeted to distinct foci which overlap with DAPI dense foci. (**D**) Immunoblot analysis demonstrating knockdown of CFDP1 protein levels in CFDP1 siRNA treated NIH3T3 cells. (**E,F**) Chromosome segregation defects in *Cfdp1* conditional knockout Mouse embryonic fibroblasts (MEFs). Immunofluorescence analysis for tubulin in uninduced MEFs (**E**) and 4-Hydroxy Tamoxifen induced (**F**) MEFs. DNA is visualized using DAPI. (**G**) Representative immunofluorescence staining for tubulin demonstrating multi-pole spindle defect in NIH3T3 cells treated with CFDP1 siRNA.

Figure S3. CFDP1 localization after nocodazole washout and NLS mapping of CFDP1 protein. (**A,B**) Representative immunofluorescence analysis demonstrating

CFDP1 localization at microtubule nucleation sites in NIH3T3 cells recovering from Nocodazole washout. Cells were fixed after 1 minute and stained for CFDP1 and Tubulin. DNA was visualized using DAPI. (**C**) cNLS Mapper prediction for a monopartite nuclear localization signal (NLS) in mouse CFDP1 protein. Position of NLS amino acids is highlighted (red) within the protein sequence.

Supplementary Text

Generation of animal models

Cfdp1-KO targeting vector was generated by sub-cloning a ~9.6 Kb genomic region from a positively identified BAC clone using a homologous recombination-based technique. The short homology arm (SA) extends 1.2 Kb from the 3′ end of *Cfdp1* exon 1 while the long homology arm (LA) begins before the start codon (ATG) at the 5′ end of *Cfdp1* exon 1 and is ~ 8 Kb long. *Cfdp1* deletion was generated by inserting a *LacZ - Neo* cassette after the start codon replacing ~376 bp within the *Cfdp1* exon 1 coding sequence. Gene targeting of *Cfdp1* in 129/Sv – derived D3 embryonic stem cells (ES) were carried out using homologous recombination. Targeted ES colonies were identified by Southern Blot hybridization of genomic DNA digested by *PstI*. A 329 bp probe based in intron 1 was used in the hybridization assay as an external probe to distinguish the wild type band (~ 5.1 Kb) and the mutant band (~3.8 Kb). Chimeric mice were generated by injecting *Cfdp1* targeted ES cell lines into C57BL/6 blastocysts. Male and female *Cfdp1* KO mice were maintained as heterozygotes.

Cfdp1 conditional KO targeting vector was generated from a 10.57 Kb genomic region sub-cloned from a BAC clone. The long homology arm (LA) extends 5.77 Kb 5′ to the location of lox P cassette inserted 1.84 Kb 5′ to the start of *Cfdp1* exon 1, while the short homology arm (SA) is 2.47 Kb in length and extends 3′ to exon 1. A *LoxP*/*FRT* flanked-Neo cassette was inserted 341 bp 3′ to *Cfdp1* exon 1. The target region excised upon *Cre* recombinase expression is 2349 bp long and includes the entire exon 1 coding region of *Cfdp1*. *Not I* linearized targeting vector was electroporated into BA1 (C57BL/6 X 129 SvEv) (Hybrid) ES cells. Recombinants were identified after G418 selection using PCR analysis and Southern blot analysis. Targeted hybrid ES cells were microinjected into C57BL/6 blastocysts and resulting chimeras were mated with C57BL/6 *FLP* mice for Neo Cassette removal. Both male and female *Cfdp1* conditional KO mice were maintained as heterozygotes and bred to homozygosity for mating experiments.

Cfdp1 KO and *Cfdp1* conditional KO alleles were identified by PCR genotyping in tail lysates generated from embryos and adult mice. Genotyping of blastocysts and preimplantation embryos were performed in lysates generated by incubating the samples at 98ºC for 10 min (10μl PBS diluted 1:1 with water). *Cfdp1* KO allele was identified using wildtype (WT) and Knockout (KO) primers (Table S1), which amplify a 479 bp genomic region and a 345 bp genomic region respectively. *Cfdp1* conditional knock out mice were identified using primers NDEL2 and NDEL1, which amplify a 570 bp genomic region indicating somatic *Neo* deletion and the presence of floxed exon1. Cre mediated recombination of the floxed allele was identified by a 3-primer strategy (NDEL2; NDEL1; CFDP IND F) which amplifies a 198bp genomic region indicative of a conditional knockout event for *Cfdp1* exon1.

Blastocyst Culture

E3.5 blastocysts were obtained from crosses between mice heterozygous for *Cfdp1* KO allele and grown on gelatinized cover slips in Embryonic Stem cell media (KnockOut DMEM) without Leukemia Inhibitory Factor (LIF) at 37°C for a total of 5 days. Blastocyst outgrowths were imaged by phase contrast microscopy or used for histology studies.

Mitotic chromosome isolation

Cells were harvested by mitotic shake-off after Nocodazole treatment as described in main methods. After centrifugation at 500 g for 10 min, cells were washed with cold PBS and suspended in 75 mM KCl. Cells were left on ice for 20 min and spun down at 1500 g for 5 min at 4ºC and homogenized in disruption buffer (10 mM Tris-HCl, pH 7.4, 120 mM KCl, 20 mM NaCl, 0.1% Triton X-100, 2 mM $CaCl_2$, 1X Protease inhibitors) by passing through a 26-gauge needle five times. Cell lysates were centrifuged at 6000 rpm for 5 min and supernatant retained. Chromosome-enriched pellets were washed once more with disruption buffer and lysed in SDS-PAGE sample buffer for immunoblot analysis.

Flow Cytometry data analysis

Flow cytometry runs were processed by FlowJo (Flowjo LLC) using a Univariate model. Events were manually gated for DNA content. After excluding debris and doublets, single cell gated population was displayed as a histogram revealing the percentage of cells in G1, S and G2/M phases using Gaussian curves to fit each cell cycle phase. Data presented were obtained from at least three independent experiments each of which were repeated in triplicates with similar results.

Complex mixture ID analysis by LC-MS/MS method

For Mass Spectrometry analysis, denatured samples were run on a 4-20% gradient acrylamide gel. Entire protein mixture was allowed to enter the resolving part of the acrylamide gel to a distance of ~1 cm. Gel was stained with QC Colloidal Coomassie Stain (Bio Rad) and gel fragments containing proteins were cut and subjected to complex mixture ID analysis. Samples were run on the Orbitrap Elite Mass Spectrometer utilizing a short reverse-phase LC-MS/MS method at the University of Texas Southwestern Proteomics Core. Protein identification was carried out using Proteome Discoverer 2.2.
