# Supplementary material for: The Chromatin Protein CFDP1 Activates TPX2 and Promotes Chromosomal Microtubule Nucleation and Spindle Assembly
Source: Int J Mol Sci. 2026 Jan 29;27(3):1362. doi: 10.3390/ijms27031362 (PMC12898535; doi:10.3390/ijms27031362)
Supplement: Supplementary file 1 [file ijms-27-01362-s001.zip › Table S1. Oligonucleotides and Antibodies used in study.pdf]

Table S1.

**Oligonucleotides for molecular cloning**	
mCFDP1 FL XhoI F	5’ AGTCTCGAGTAGAGGAATTCGACTCCG 3’
mCFDP1 FL BamHI R	5’ GCGGGATCCAGGTTTCATTTTGCTCAG 3’
mH2a.z FLAG XbaI F	5’ AGTTCTAGATGGACTACAAAGACGATGACGACAAGATG GCTGGCGGTAAGGCT 3’
mH2a.z HindIII R	5’ GCGAAGCTTAAACAGTCTTCTGTTGTC 3’
mCFDP1 (1-885) F	5’AGTTCTAGATGAGAGGATCGCATCACCATCACCATCACGA GGAATTCGACTC 3’
mCFDP1 (1-885) R	5’ GCGGGATCCTTAAGGTTTCATTTTGCTCAG 3’
mCFDP1 (1-450) R	5’ GCGGGATCCTTACAGCTCATCTGCTTTGAC 3’
mCFDP1 (451-885) F	5’AGTTCTAGATGAGAGGATCGCATCACCATCACCATCACGAT AAGCCTAGAGA 3’
mCFDP1 (297-597) F	5’AGTTCTAGATGAGAGGATCGCATCACCATCACCATCACAAT GCAAGGAAGAAG 3’
mCFDP1 (297-597) R	GCGGGATCCTTAAGCCTGCGGTTTTTCTCT 3’
mCFDP1 (654) F	5’AGTTCTAGATGAGAGGATCGCATCACCATCACCATCACGG TATGAGCAGCCTC 3’
**Oligonucleotides for genotyping**	
CFDP1 WT F	5’ ACTTCTCCACATCGGACGAG 3’
CFDP1 WT R	5’ ATGGGTAGCTCCTGCAAAGA 3’
CFDP1 KO F	5’ GAT CGGCCATTGAACAAGAT 3’
CFDP1 KO R	5’ ATACTTTCTCGGCAGGAGCA 3’
NDEL 1	5’ GGACGATGGGTAGCTCCTGCA 3’
NDEL 2	5’ CTCACTCGAGACTCTCCCGGAC 3’
CFDP IND F	5’ CGCGGCTTGATGAGGAGATG 3’
**Short interfering RNA (Dharmacon/Horizon scientific)**	
ON-TARGETplus Cfdp1 siRNA	Cat# L-043538-01-0005
ON-TARGETplus Non-targeting Pool	Cat# D-001810-10-05

REAGENT or RESOURCE	SOURCE	IDENTIFIER
**Antibodies**		
Rabbit Polyclonal Anti-CFDP1 Antibody	*In-house*	Diekwisch and Luan, 2002
Mouse Monoclonal Anti-CFDP1 Antibody	Sigma	SAB-2702263
Rabbit MAD1 polyclonal antibody	Thermo Fisher Scientific	Cat# PA5-28991, RRID:AB_2546467
Rabbit Anti-Mouse CENP-A, Clone C51A7	Cell Signaling Technology	Cat# 2048, RRID:AB_1147629
Rat Anti-Tubulin Monoclonal Antibody, Clone YOL1/34	Abcam	Cat# ab6161, RRID: AB_305329
Mouse Anti-Cyclin A Monoclonal Antibody, Clone E23.1	Abcam	Cat# ab38, RRID: AB_304084
Rabbit Monoclonal Anti-Cyclin D1 [EPR2241]-C-terminal antibody	Abcam	Cat# ab134175, RRID: AB_2750906
Mouse Monoclonal Anti-Cyclin B1 Antibody (GNS1)	Thermo Fisher Scientific	Cat# MA5-14319, RRID: AB_10987286
Rabbit Monoclonal Anti-CDC20(D6C2Q) Antibody	Cell Signaling Technology	Cat# 14866, RRID: AB_2715567
Rabbit Anti-MAD2 Polyclonal Antibody	Thermo Fisher Scientific	Cat# PA5-21594, RRID: AB_11154021
Mouse Monoclonal Anti-PCNA (PC10) Antibody	Cell Signaling Technology	Cat# 2586, RRID: AB_2160343
Mouse Anti-Actin, beta Monoclonal Antibody, Clone ACTN05(C4)	Abcam	Cat# ab3280, RRID: AB_303668
Rabbit Monoclonal Anti-Bub3 (D8G6) Antibody	Cell Signaling Technology	Cat# 8194, RRID: AB_10859892
Rabbit Polyclonal Anti-beta Tubulin Antibody	Abcam	Cat# ab6046, RRID: AB_2210370
Rabbit Polyclonal Anti-Histone H2A Antibody-ChIP Grade	Abcam	Cat# ab18255, RRID: AB_470265

Rabbit Polyclonal Anti-Histone H3 Antibody – Nuclear Loading Control and ChIP Grade	Abcam	Cat# ab1791, RRID: AB_302613
Rabbit Polyclonal Anti-INCENP Antibody	Abcam	Cat# ab12183, RRID: AB_298914
Rabbit Polyclonal Anti-Aurora B Antibody	Abcam	Cat# ab2254, RRID: AB_302923
Mouse Monoclonal Anti-TPX2 Antibody [18D5-1]	Abcam	Cat# ab32795, RRID: AB_778561
Mouse Monoclonal ANTI-FLAG M2 Antibody	Sigma-Aldrich	Cat# F1804, RRID: AB_262044
Alexa Fluor 568 Donkey Anti-Mouse IgG (H+L)	Thermo Fisher Scientific	Cat# A-10037, RRID: AB_2534013
Alexa Fluor 594 Donkey Anti-Mouse IgG (H+L)	Thermo Fisher Scientific	Cat# A-21203, RRID: AB_141633
Alexa Fluor 488 Chicken Anti-Rabbit IgG (H+L)	Thermo Fisher Scientific	Cat# A-21441, RRID: AB_141735
Alexa Fluor 647 Goat Anti-Rabbit IgG (H+L)	Thermo Fisher Scientific	Cat# A-32733, RRID: AB_2633282
Anti-Mouse IgG, HRP-linked Antibody	Cell Signaling Technology	Cat# 7076, RRID: AB_330924
Anti-Rat IgG, HRP-linked Antibody	Cell Signaling Technology	Cat# 7077, RRID: AB_10694715
Anti-Rabbit IgG, HRP-linked Antibody	Cell Signaling Technology	Cat# 7074, RRID: AB_2099233
Rabbit Polyclonal Anti-Laminin Antibody	Abcam	Cat# ab30320, RRID: AB_775970
Rabbit Polyclonal GATA4 Antibody	Abcam	Cat# ab61170, RRID: AB_941726
Rabbit Anti-SNAIL Polyclonal Antibody	Abcam	Cat#63371, RRID: AB_2191754

Chicken Polyclonal Anti-EED Antibody	Abcam	Cat# ab14294, RRID: AB_2262068
