# Supplementary figures and images for: The Chromatin Protein CFDP1 Activates TPX2 and Promotes Chromosomal Microtubule Nucleation and Spindle Assembly
Source: Int J Mol Sci. 2026 Jan 29;27(3):1362. doi: 10.3390/ijms27031362 (PMC12898535; doi:10.3390/ijms27031362)

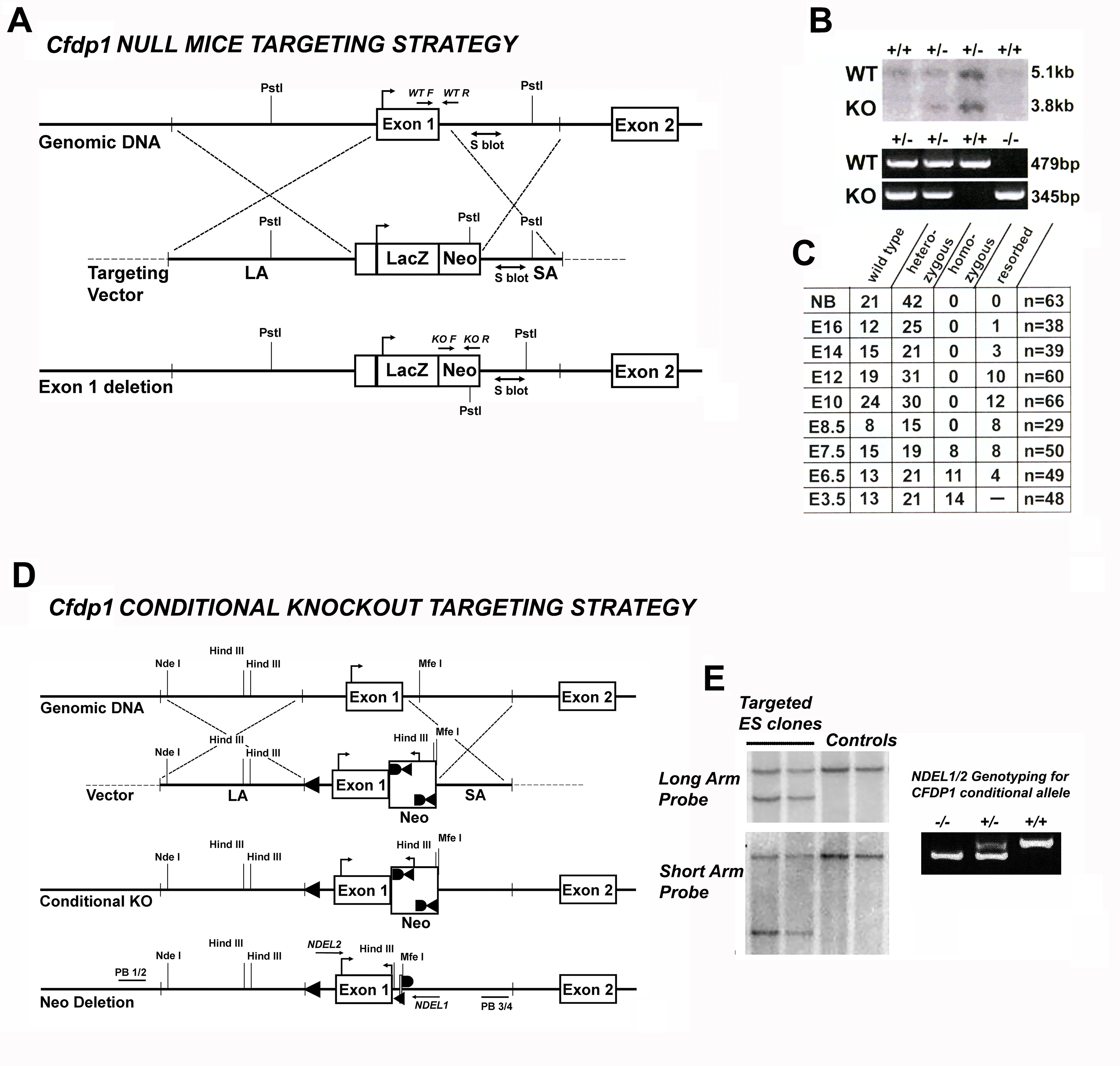

Supplement: Supplementary file 1 [file ijms-27-01362-s001.zip › Supplementary Figure S1.tif]

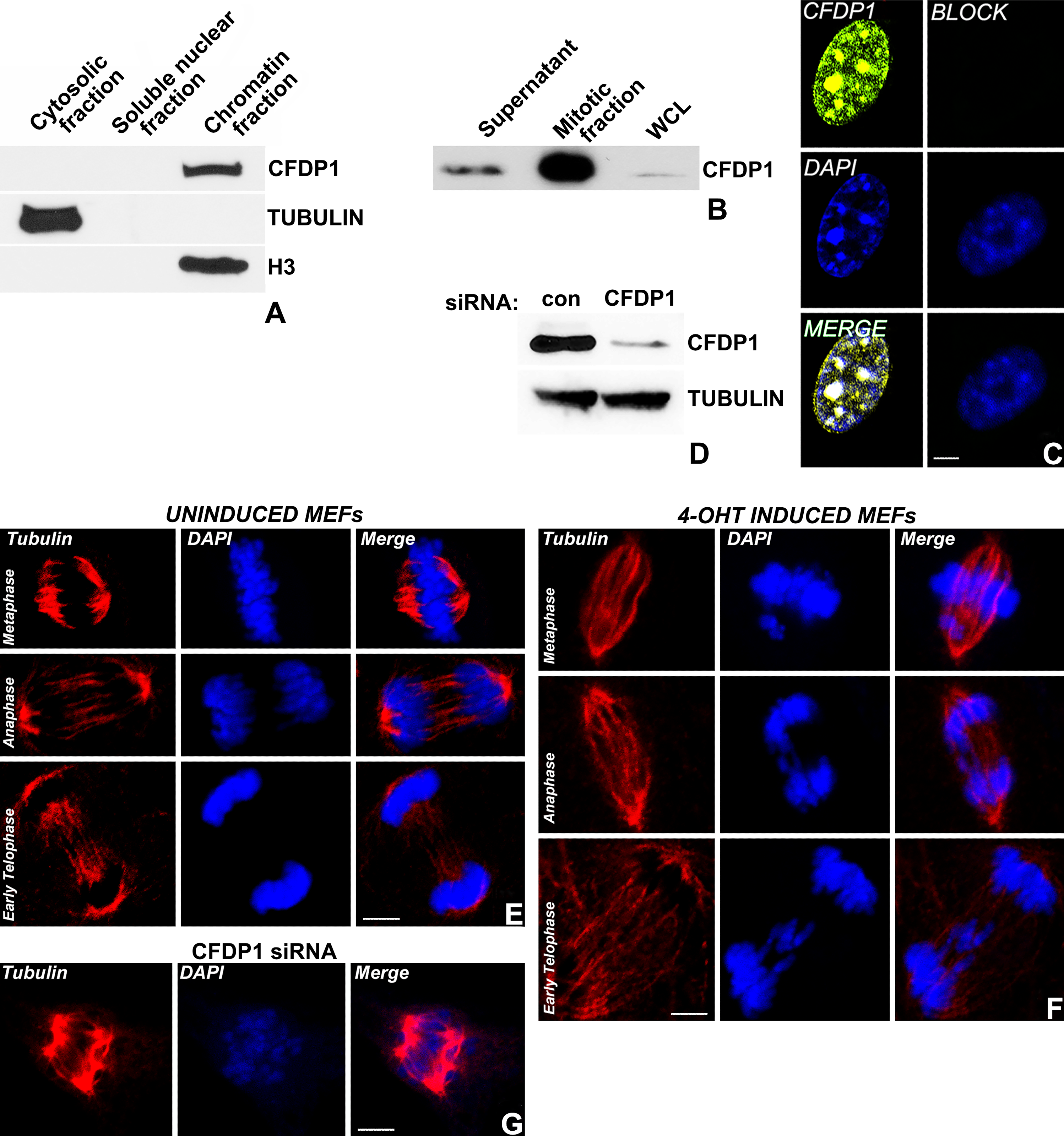

Supplement: Supplementary file 1 [file ijms-27-01362-s001.zip › Supplementary Figure S2.tif]

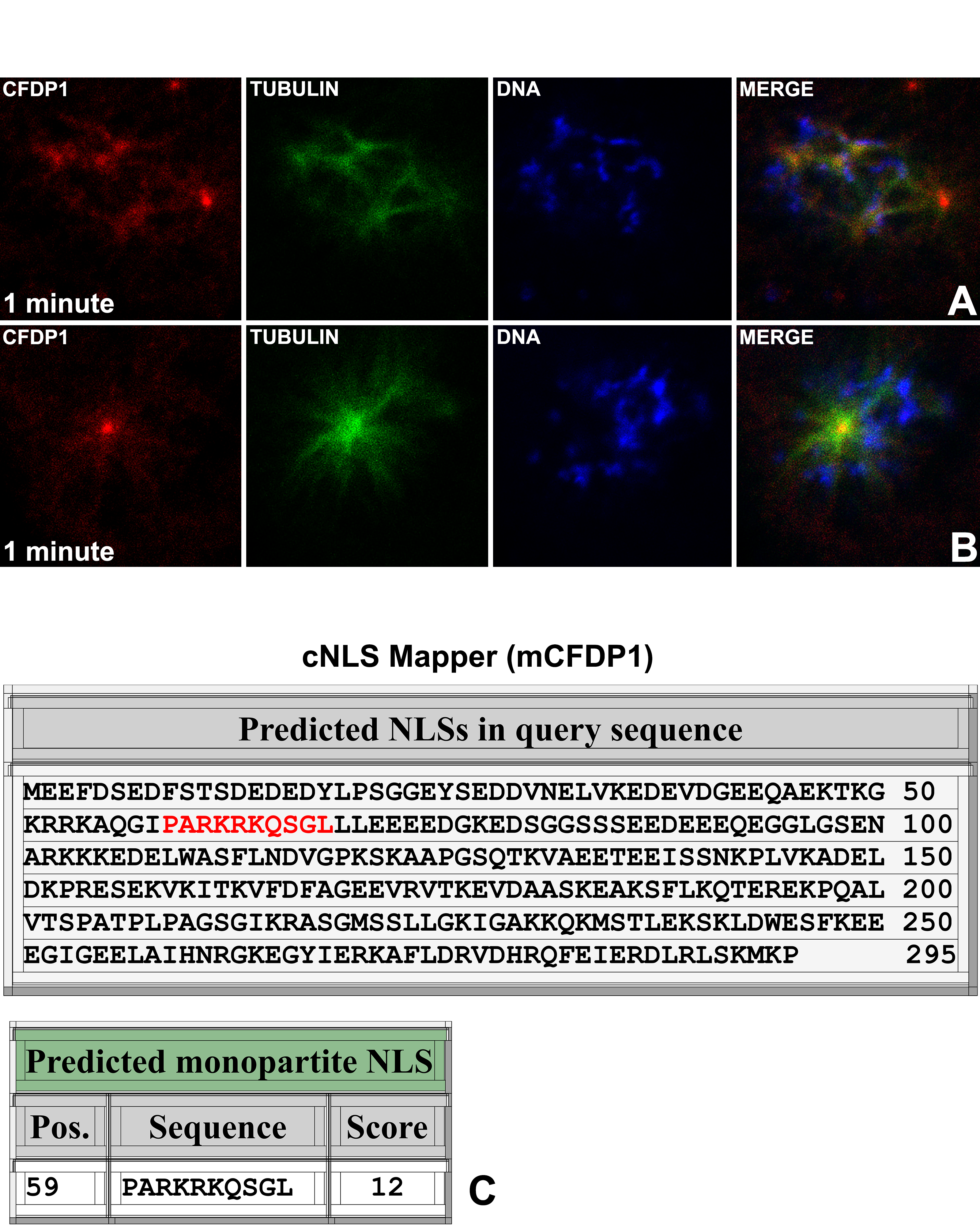

Supplement: Supplementary file 1 [file ijms-27-01362-s001.zip › Supplementary Figure S3.tif]
